# Objective and subjective measures of neighborhood environment (NE): relationships with transportation physical activity among older persons

**DOI:** 10.1186/s12966-015-0276-3

**Published:** 2015-09-15

**Authors:** Ma Shwe Zin Nyunt, Faysal Kabir Shuvo, Jia Yen Eng, Keng Bee Yap, Samuel Scherer, Li Min Hee, Siew Pang Chan, Tze Pin Ng

**Affiliations:** Gerontology Research Programme, Department of Psychological Medicine, Yong Loo Lin School of Medicine, National University of Singapore, Kent Ridge, Singapore; Centre for Sustainable Asian Cities, National University of Singapore, Singapore, Singapore; Department of Rehabilitation Care, National University Health System, Kent Ridge, Singapore; Department of Geriatric Medicine, Alexandra Hospital, south-western, Singapore; Department of Medicine, Yong Loo School of Medicine, National University of Singapore, Kent Ridge, Singapore; Department of Medicine, University of Melbourne, Melbourne, Australia; Cardiovascular Research Institute, National University Heart Center, Kent Ridge, Singapore; Department of Mathematics & Statistics, Faculty of Science, Technology & Engineering, La Trobe University, Melbourne, Australia; Center for Liveable Cities, Ministry of National Development, Singapore, Singapore; Yong Loo Lin School of Medicine, Department of Psychological Medicine, National University Hospital, National University of Singapore, 5 Lower Road, Kent Ridge, 119074 Singapore

**Keywords:** Built environment, Transportation physical activity, Elderly, Neighborhood environment walkability scale (NEWS), Geographical information system (GIS), Structural equation model (SEM)

## Abstract

**Background:**

This study examined the associations of subjective and objective measures of the neighbourhood environment with the transportation physical activity of community-dwelling older persons in Singapore.

**Method:**

A modified version of the Neighborhood Environment Walkability Scale (NEWS) and Geographical Information System (GIS) measures of the built environment characteristics were related to the frequency of walking for transportation purpose in a study sample of older persons living in high-density apartment blocks within a public housing estate in Singapore. Relevant measured variables to assess the complex relationships among built environment measures and transportation physical activity were examined using structural equation modelling and multiple regression analyses.

**Results:**

The subjective measures of residential density, street connectivity, land use mix diversity and aesthetic environment and the objective GIS measure of Accessibility Index have positively significant independent associations with transportation physical activity, after adjusting for demographics, socio-economic and health status.

**Conclusion:**

Subjective and objective measures are non-overlapping measures complementing each other in providing information on built environment characteristics. For elderly living in a high-density urban neighborhood, well connected street, diversity of land use mix, close proximity to amenities and facilities, and aesthetic environment were associated with higher frequency of walking for transportation purposes.

## Introduction

Successful “aging in place” involves enabling the elderly to be physically active and independent in performing instrumental activities of daily living for meaningful social engagement within their own community and neighborhood. The physical characteristics of the neighborhood built environment play an important intervening role in supporting or limiting physical activity and functional independence of elderly people living in their community [1, 2].

Recent research examining the links between characteristics of the physical neighborhood environment (NE) and the frequency and duration of transport-related physical activity (PA) (e.g., walking to the grocery store) within urban communities have variously used perceived measures obtained by interviews and geospatial data based on geographical information system (GIS) analyses of archival maps (for a review of studies, see study by Lin, et al., 2010) [3]. The development and evaluation of measures of the built environment are still at a relatively early stage. There remains much to be explored, especially from a public health point of view, about the relevance and utility of various elements of the built environment to different domains of physical activity (transportation and leisure) and other functional outcomes for various population groups including the elderly [3].

The extent to which objective and subjective measures of the neighbourhood environment overlap or complement each other in influencing the level of physical activity and functional independence is not well elucidated. Prior studies [4, 5] indicate a poor level of agreement between objective and perceived measures of the built environment. Both objective and perceived measures evaluated in the same model showed independent associations with physical activity, thus suggesting that the same aspects of the built environment should be ascertained with both objective and subjective measures. Few studies have explored the individual, combined and relative contributions of subjectively or objectively measured attributes of the built environment to physical activity by the elderly.

Studies show that GIS measures of the amount of automobile traffic and number of commercial establishments in the neighborhood were reportedly associated with increased levels of overall walking activity [6]; and GIS measures of land-use diversity was reportedly associated with greater independence in instrumental activities of daily living [1]. In Japan, perceived measures of good bicycle lanes, non-ownership of household motor vehicles and access to exercise facilities were found to be significantly associated with higher levels of transportation physical activity among seniors, whereas access to public transportation was not [7]. This suggests that the relevance of a wide range of built environment characteristics to transportation physical activity and functional independence may differ among countries, according to the level and socio-cultural characteristics of their infrastructural development.

Singapore is characterized by its small land size (718.3 km^2^ in 2014) and high density population density (7615 persons/km^2^ in 2014), with 82 % of the population residing in high rise apartments [8]. With its exceptionally rapid rate of population ageing, approximately 30 % of the total residential population of Singapore will be above 65 years of age in 2050, up from 10 % in 2010. The authorities have in recent years begun to pay more attention to developing an urban infrastructure that provides an elder-friendly living environment in support of active aging.

The aim of this study was to assess the use of both perceived assessments and objective GIS measures of the neighborhood environment to examine their independent and combined associations with transportation physical activity among community dwelling elderly in Singapore.

## Methods

### Study population

A total of 402 older persons (≥55 years of age) who were resident for at least 5 years in a neighborhood located in three public housing precincts in the South Central region of Singapore participated in this study. They were among 2802 participants in an ongoing population-based longitudinal ageing cohort study (Singapore Longitudinal Aging Study Wave 2) conducted in 2011 and 2012. We invited one elderly resident per residential address to participate in the study. Older persons with severe physical disability and communication difficulties were excluded. Trained research nurses visited their homes to conduct face-to-face interviews. The study was approved by the National University of Singapore Institutional Review Board (NUS-IRB), and all study participants gave informed consent.

### Measurements

The subjective measure of the perceived neighborhood environment used an adapted version of the Neighborhood Environment Walkability Scale (NEWS) [9]. It comprises 67 items which are grouped into 8 factors:

a Residential density—types of residences in the neighborhood
b Land use mix—diversity (“Stores, facilities, and other things in your neighbourhood”)

was assessed by reports of facilities and amenities in the neighborhood, such as “About how long would it take to get from your home to the nearest businesses or facilities listed below if you walked to them?” and “How much it influences your participation in doing activities”

c Street connectivity—includes streets in the neighborhood, places for walking and cycling and neighborhood surroundings
d Land use mix—access (Access to services) such as “I can do most of my shopping at local stores”, “It is easy to walk to a transit stop (bus, train) from my home” (1 = strongly disagree, 2 = somewhat disagree, 3 = somewhat agree, 4 = strongly agree)
e Infrastructure—places for walking and cycling
f Aesthetics (Neighborhood surroundings)
g Traffic safety
h Safety from crime [10, 11].

Objective measures of the neighborhood environment was based on Geographical Information System (GIS) variables measured in Euclidean or straight-line distances buffer within 500 m of the centroid of a neighborhood using the software ArcGIS® version 10 [12]:

a Street connectivity—based on the number of true intersections within a given area, was defined by the number of street links divided by the number of street nodes within the buffer area.
b Residential density—the number of dwelling units was divided by the land area in residential use within the area
c Land use mix—the distribution of development across five uses (residential, commercial, industrial, recreation and other) is assessed to measure the land use mix.
d Public park density—obtained by dividing the total area of public parks by the total area of the buffer and multiplying by 100

The Walkability Index was in turn derived as a composite value of combinations and weights of individual built environment characteristics, including residential density, street connectivity, land-use mix [13–16]. Since urban Singapore is characterized by a mixed land-used and compact urban environment, the net retail area was omitted in this study. Because of the unavailability of GIS layer of walkable paths within the study area, the impact of street networks is considered to be homogenous. “Residential lot coverage” and “street density” were used as proxy variables respectively for residential density and street connectivity. Therefore, the modified walkability index included residential lot coverage, street density and land-use mix. The built environment characteristics were measured in numerical values by ArcGIS® and Z-scores were calculated after arbitrarily dividing the whole study area into 18 zones (study area units). The constituent variables of walkability index, i.e. residential density, street density and land-use mix, were calculated based on the formulas provided in the Neighborhood Environment for Active Transport-Geographic Information Systems(NEAT-GIS) protocol [17]. Specifically,

Walkability index = Residential lot coverage + 1.5 × street density + land use mix

The Accessibility Index was assessed by measuring the walking access to 30 types of community service and amenity destinations to which proximity could plausibly encourage residents to walk more for leisure or transport [18]. Accessibility index is calculated for the 18 individual zones based on the multiplication of the sum of building weight within each zone and the residential density:

Accessibility index = building weights of the zone × residential density.

Both the Walkability and Accessibility Indexes were categorized into 3 levels, namely low, medium and high.

Physical activity (walking for transportation purpose); The study participants were also asked “How often do you walk from your house to (nearest) various types of businesses or facilities” with response ranging from never to daily. The scores of all items were summed and a higher scores denoting more frequently walking for transport. Transportation physical activity was the primary outcome of the analysis.

Covariates The study participant’s self-rated health was reported as ‘excellent’ , ‘very good’ , ‘good’ , ‘poor’ , or ‘very poor’. Physical Performance: gait and balance were measured with the performance oriented mobility assessment (POMA) tool [19]. Static sitting balance (rising from the sitting position without using hands) was assessed using graded scores by the need for assistance and the number of attempts; standing balance was assessed within the first 5 s after the subject’s sternum was gently pushed by the examiner, and when stance was stabilized. Staggering or excessive sway of the subject was examined with the subject standing and eyes closed. Steadiness and continuity of steps were observed with the subject turning in a complete 360° circle. The gait assessment was performed with the subject walking 6 m and returning quickly to the starting point, noting the ability to initiate walking and any hesitancy, step height and length, the lack of symmetry or inability to clear the floor, step continuity, deviation in the path and walking stance. The POMA scores for balance and gait were tallied separately using standard scoring criteria. Socio-demographic data included age, gender, ethnicity, educational attainment and housing type.

### Data analysis

Structural Equation Models (SEM) based on robust maximum-likelihood estimation were used to perform regression analysis with observed (measured or manifest) data and factor analysis with latent variables simultaneously, given the complex relationships among the considered variables. Subjective and objective neighborhood environment (NE) measurement constructs and socio-economic and physical health status were treated as latent variables, as they were not directly measured but inferred from the observed variables. Raw scores of all measured variables in every factor of objective and subjective NE measures were used in analysis. The subjective and objective NE measures were linked with a covariance given their similar nature in explaining the level of transportation physical activities. In Confirmatoty Factor Analysis, a measurement model was built for each subjective and objective measure factors. The first indicator of each latent variable was fixed at 1.0, in order to create a metric scale. Indicators which were not significantly correlated to the latent variables were removed from the model.

Multiple Regression Models were used to examine the association of subjective and objective measures of the built environment characteristics (independent variables) with transportation physical activity (dependent variables). In stepwise models, socio-economic variables, self-rated health status, and POMA balance and gait scores were analyzed in the base model. Next, we added subjective or GIS measures of the built environment in separate models (1a and 1b). Finally, we added both subjective and objective measures together to the base model (Model 2). Adjusted R squared changes from base model to full model (Model 2) were identified.

Data analysis was carried out with Stata MP version 13.0 [20]. The root mean square errors of approximation (RMSEA) were generated as the default index of model fit [21, 22]. All statistical tests were performed at 5 % level of significance.

## Results

The sample characteristics are depicted in Table 1. The mean age of the study participants was 69.1 years. They were dominantly female (60.7 %), and of Chinese ethnicity (83.1 %). About 71.4 % had primary or no formal education, and approximately 42 % lived in 1 or 2 room public housing apartments. The majority of the subjects rated their health as good, very good or excellent (81.1 %).

**Table 1:** Sample characteristics (*n* = 402)

Variables		Number	Percent
Gender	Male	157	39.1
	Female	244	60.7
Age	Mean (SD)	69.13	(8.53)
	> = 65	265	65.9
	<65	137	34.1
Ethnicity	Non-Chinese	68	16.9
	Chinese	334	83.1
Education	No formal/Primary	287	71.4
	Secondary & Above	113	28.1
Housing status	1 or 2 Rooms	169	42.0
	3 rooms	143	35.6
	4 or 5 Rooms	90	22.4
Self-rated health status	Poor	4	1.0
	fair	112	27.9
	Good	212	52.7
	Very good	62	15.4
	Excellent	12	3.0
POMA Balance score	Mean (SD)	15.67	(1.33)
POMA Gait score	Mean (SD)	11.70	(0.94)
GIS Built environment measures			
Walkability index	Low	48	11.9
	Medium	255	63.4
	High	96	24.4
Accessibility index	Low	137	34.1
	Medium	165	41.0
	High	99	24.6
Physical activity			
Walking for transportation	Mean (SD)	25.16	(0.58)

The confirmatory factor analyses showed that most of the manifest variables loaded satisfactorily on their respective latent variables, representing transportation physical activity and eight subjective BE measures, namely residential density, well connected streets in the neighborhood (street connectivity), land use mix access, infrastructure walk cycle, aesthetics, traffic safety, crime safety and land use density. Details of the CFA are described in the Appendix, and results of factor analysis are shown in Tables 3, 4, and 5.

**Fig. 1:**
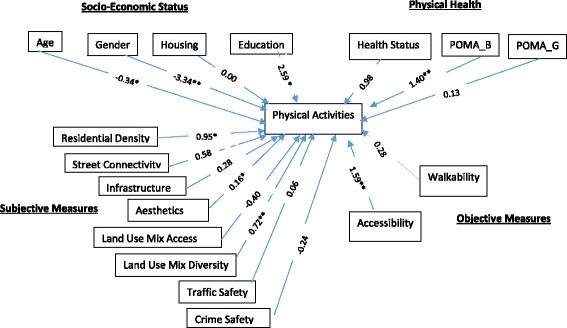
Association of Physical Activities of Community Dwelling with Socio-Economic and Health Status factors and Built Environment (Subjective and Objective Measures) Note: Multiple regression model adjusted with socio-economic and health status factors (coefficient* *p* < 0.001 and coefficient**p < 0.05)

In multiple regression analyses, we ascertained whether the eight subjective NE measures and two objective GIS indices (walkability and accessibility) were significantly associated with transportation physical activity (See Fig. 1). Covariates in the regression analyses included socio-economic variables (age, gender, housing type and educational attainment), and physical health (health status, POMA Balance Score and POMA Gait Score). To facilitate comparison, four models were built. These included the base model (with socio-economic variables only), base model and subjective measures of built environment (Model 1a), base model and objective measures (Model 1b) and finally the full model (Model 2) comprised all the base model, subjective and objective variables (Table 2).

**Table 2:** Association of physical activities of community dwelling with built environment (Subjective and Objective Measures) after adjusted with socio-economic and health status (Multiple regression Model)

	Coefficient	95 % CI		*p*-value	Adjusted R2
Base model variables:					0.19
Age	−0.34	−0.49	−0.19	<0.001	
Gender	−3.34	−5.76	−0.91	0.007	
Housing type	0.00	−0.01	0.01	0.56	
Education	2.59	1.18	4.01	<0.001	
Self- rated health status	0.98	−0.57	2.53	0.21	
POMA Balance score	1.40	0.40	2.39	0.006	
POMA gait score	0.13	−1.27	1.54	0.85	
Model 1a: Base model + subjective measures					0.43
Resident density	1.07	0.58	1.57	<0.001	
Street connectivity	0.69	0.05	1.34	0.04	
Land use mix-access	−0.42	−0.93	0.10	0.11	
Land use mix-diversity	0.72	0.18	1.25	0.01	
Infra-structure for walking and cycling	0.22	−0.23	0.67	0.34	
Aesthetics	0.17	0.12	0.21	<0.001	
Traffic safety	0.02	−0.31	0.35	0.90	
Crime safety	−0.23	−0.66	0.21	0.31	
Model 1b: Base model + objective measures					0.24
GIS Walkability	1.05	−1.06	3.15	0.33	
GIS Accessibility	4.28	2.61	5.94	<0.001	
Model 2: Base model + subjective + objective measures					0.43
Resident density	0.95	0.44	1.46	<0.001	
Street connectivity	0.58	−0.07	1.24	0.08	
Land use mix-access	−0.40	−0.92	0.13	0.14	
Land use mix-diversity	0.72	0.18	1.26	0.009	
Infra-structure for walking and cycling	0.28	−0.17	0.73	0.22	
Aesthetics	0.16	0.11	0.21	<0.001	
Traffic safety	0.06	−0.27	0.39	0.72	
Crime safety	−0.24	−0.68	0.20	0.29	
GIS Walkability	0.28	−1.61	2.17	0.77	
GIS Accessibility	1.59	0.02	3.15	0.05	

The base model (adjusted R-squared 0.19) showed that older (β = −0.34, *p* < 0.001), female subjects (β = −3.34, *p* < 0.05) were more likely to have a lower level of transportation physical activity and elderly with higher education (β = 2.59, *p* < 0.001) and better physical performances (β = 1.40, *p* < 0.05) were more likely to have a higher level of transportation physical activity. Model 1a (adjusted R-squared 0.43) showed R-squared change of 0.24, suggesting that subjective BE measures gave a substantially larger contribution to the level of transportation physical activity than objective measures, which was associated with a R-squared change of 0.05 in Model 1b (R-squared = 0.24). The final full model (Model 2) indicated that combining subjective and objective measures did not substantially increase the total R-squared. Among the eight subjective measures, resident density (β = 0.95, *p* < 0.001), land-use mix density (β = 0.72, *p* < 0.05) and aesthetic environment (β = 0.17, *p* < 0.001) were significant in explaining the level of transportation physical activity. Of the objective GIS measure, only the Accessibility Index (β = 1.59, *p* < 0.05) was significant.

## Discussion

We found in this study of older persons living in a high-density urban neighborhood in Singapore that residential density, diversity of land use mix, close proximity to amenities and facilities, and aesthetics were demonstrably associated with higher frequency of walking for transportation purposes, such as going to and from shopping. Our findings are consistent with other reports that residents are more likely to engage in transportation-related physical activity if they live in neighbourhoods with higher-density housing, easier access to a range of destinations, well connected street networks with aesthetic environment and a mix of land-use zones [23, 24].

Our study further provides insights on the use of subjective and objective measures of neighbourhood environment characteristics in relation to transportation physical activity among older adults. Both subjective and objective measures of accessibility reflecting proximity to community services and amenity destinations that could plausibly encourage residents to walk more for leisure or transport [18] were used in this study. Interestingly, GIS measure of accessibility was demonstrated to be significantly associated with transportation physical activity, whereas perceived measure of accessibility was not. This suggests that the objective measure was a more valid measure of accessibility than the subjective measure. Conversely, the GIS measure of walkability (constituted by residential density, street connectivity and land-use mix) was not significantly associated with transportation physical activity, whereas subjective measures of perceived street connectivity, land use mix-diversity and aesthetic environment were significantly associated. They thus appear to provide additional information predicting the probability of more frequent transportation physical activity that were not measured by the objective measure. In agreement with prior studies [3, 4], there were no uniformly strong correlations between objective and perceived measures for different built environment attributes, suggesting that the same aspect of the built environment may be measured by one measure that is not fully measured by the other. Our study suggests that subjective and objective measures complement each other in providing information on built environment characteristics.

In this study, we also confirmed the manifest variables which were relevant for the measuring built environment attributes of high-density urban neighborhoods typical for over 80 % of the population in Singapore. Uniquely, these public housing estates have wet markets and supermarket for grocery, and almost all elderly do not own cars. Therefore, dead end streets, shopping at local stores and difficulty parking were not relevant measurement items in the Singapore context. This suggests that the measurement and modification of built environment characteristics for improved transportation physical activity and mobility are unique to a location, and need to be relevant to the level and socio-cultural context.

A strength of this study is the use of both subjective and objective measures of built environment for small geographic units. The results in this study is generalizable to many neighborhoods in Singapore, since the study population and geographical site is typical of many older public housing estates. However, further studies are required to determine the variations in the influence of the neighborhood environment on older persons’ mobility by different housing and environmental design types.

There are limitations in this study. Because of its cross sectional design, the causality of the observed associations should be cautiously interpreted. The accuracy of questionnaire responses by elderly people may be subject to recall bias and inaccuracy, and the self-reported data on subjective measures of the neighborhood environment and physical activity may contribute to a positive response bias favoring a closer association of two self-reported measures. Instead, actigraphy may provide a more objective measure of physical activity.

In conclusion, the important role of the physical built environment in influencing the level of transportation physical activity of older persons living in the community is firmly supported in this study. Our study provides supporting evidence to suggest that urban housing and environmental design planning that provide adequate number of facilities and amenities in close proximity to apartment blocks, and aesthetic neighborhood environment will positively influence older residents to walk more for transport, promote independent living in the community and maintain their quality of life.

## Appendix

Transportation physical Activity The manifest variables considered were carefully selected so that they were relevant to Singapore’s context, in terms of socio-demographic characteristics, culture and environmental features. A number of items in the original questionnaires and instruments were omitted before analysis. These included “shopping at local stores”, “dead end street in the neighborhood”, “car parking difficulty in shopping areas”, “interesting things to look in the neighborhood” and “exhaust fumes”. In Singapore, almost every public housing estate has wet markets and supermarkets for grocery and most of the elderly are not car owners. Therefore, dead end streets, shopping at local stores and difficulty parking are not relevant in this study. In addition, elderly residents usually do not find their neighbourhood interesting or exciting because they have been living there for many years and all housing estates are equipped with similar basic infrastructures and facilities, owing to the government’s urban planning.

Based on the SEM framework, the confirmatory factor analyses showed that most of the manifest variables loaded satisfactorily on their respective latent variables (eight subjective measures and the outcome of transportation physical activity). For transportation physical activity, (Table 3) almost all of the locations (including fast food restaurant, coffee shop, religious institutions, nearest bus stop, post office, etc.) were significantly loaded on transport physical activity. Medicinal shops were not, partly because the community-based polyclinics are providing almost all necessary health care to the elderly at public subsidized rates. The effect of senior activity center and hub could also be explained by community club and residents’ committee.

**Table 3:** Factor analysis of transportation physical activity

Measurements	Coefficient	95 % C.I		*P* value
Provision shop	1 (Constrained)			
Wet market	1.24	0.74	1.75	<0.001
Supermarket	1.67	1.05	2.30	<0.001
Hardware store	0.58	0.34	0.82	<0.001
Laundry/dry cleaners	0.13	0.05	0.21	0.002
Clothing shops	0.39	0.18	0.59	<0.001
Hairdresser/barber shop	0.52	0.24	0.79	<0.001
Hawker center	1.76	1.10	2.43	<0.001
Coffee shop	1.73	1.06	2.39	<0.001
Fast food restaurant	0.70	0.40	1.01	<0.001
Non-fast food restaurant	0.89	0.54	1.24	<0.001
Bank/Automated Teller Machines (ATM)	1.40	0.88	1.92	<0.001
Post office	0.83	0.50	1.16	<0.001
Library	1.00	0.62	1.39	<0.001
Religious institutions	0.88	0.50	1.26	<0.001
Elementary school	0.80	0.46	1.13	<0.001
Other schools	0.24	0.10	0.38	<0.001
Book store	0.27	0.13	0.40	<0.001
Video store	0.12	0.04	0.20	<0.001
Movie theatre	0.18	0.09	0.26	<0.001
Medicinal shops	0.14	−0.09	0.37	0.239
Clinics/dentals	0.39	0.20	0.58	<0.001
Nearest bus stop	1.42	0.87	1.97	<0.001
Nearest Mass Rapid Transport (MRT) station	1.58	1.00	2.16	<0.001
Recreational park	0.73	0.31	1.16	<0.001
Community club/Residents’ Committee	0.76	0.38	1.14	<0.001
Swimming pools	0.36	0.18	0.55	<0.001
Gym or fitness facility	0.64	0.28	1.00	<0.001
Singapore pools	0.25	0.05	0.45	0.016
Senior activity center/club	−0.32	−0.73	0.08	0.116

CFA generated eight subjective measures of built environment, namely residential density, well connected streets in the neighborhood (street connectivity), land use mix access, infrastructure walk cycle, aesthetics, traffic safety, crime safety and land use density, and details shown in Table 4 and 5 below. In each factors, the significant manifest variables (*p* < 0.05) were identified and retained for explaining eight subjective measures of built environment The results were compared with the full list of manifest variables with the aid of Akaike and Bayesian Information Criteria. The results of the factor analyses were found to be satisfactory.

**Table 4:** Factor analysis of subjective measures of built environment

Measurements	Coefficient	95 % CI		p
Residential density (Factor 1)				
Bungalow	1 (constrained)			
Shophouses/house (1–3 stories)	1.33	1.27	1.39	<0.001
Apartment/condominium (1–3 stories)	5.94	1.53	10.36	0.008
Apartment/condominium (4–6 stories)	5.86	1.44	10.27	0.009
Apartment/condominium (7–12 stories)	43.89	13.11	74.68	0.005
Apartment/condominium (more than 13 stories)	45.51	13.88	77.14	0.005
Street Connectivity (Factor 2)				
Connecting and covered walkways	1 (constrained)			
Sufficient time for pedestrian crossing	1.20	0.94	1.46	<0.001
Nearest traffic light	0.93	0.75	1.10	<0.001
Land Use Mix Access (Factor 3)				
Store within easy walking distance	1 (constrained)			
Places within easy walking distance	0.66	0.56	0.75	<0.001
Easy to walk to bus & Mass Rapid Transport stations	0.99	0.99	1.00	<0.001
Many steps/stairs difficult to walk	0.66	0.56	0.75	<0.001
Infrastructure Walk cycle (Factor 4)				
Sidewalks on most streets	1 (constrained)			
Well-designed sidewalks	1.37	1.13	1.61	<0.001
Bicycles lanes	−2.02	−2.48	−1.55	<0.001
Sidewalks separated from streets by barriers	2.08	1.68	2.47	<0.001
Cars parked between sidewalks and main streets	0.46	0.17	0.75	0.002
Grass separaters separates roads from sidewalks	2.02	1.63	2.41	<0.001
Aesthetics (Factor 5)				
Shade for sidewalks	1 (constrained)			
Trees along the streets	1.12	0.58	1.67	<0.001
Interesting things to look while walking	0.02	−0.17	0.22	0.81
Free from litter	−0.23	−0.43	−0.03	0.02
Trees and garden landscaping	0.22	−0.01	0.46	0.06
Attractive buildings/homes	−0.21	−0.37	−0.05	0.01

**Table 5:** Factor analysis of Subjective measures (Continued)

Measurements	Coefficient	95 % CI		p value
Traffic safety (Factor 6)				
Much traffic along the streets	1 (constrained)			
Much traffic along nearby streets	1.00	0.75	1.25	<0.001
Traffic speed slow	0.94	0.69	1.19	<0.001
Traffic speed slow on most nearby streets	1.00	0.75	1.26	<0.001
Drivers exceed the posted speed limits	1.02	0.76	1.28	<0.001
Drivers stopped at pedestrians crossing	1.51	1.20	1.82	<0.001
Pedestrians crossings and signals	1.40	1.11	1.69	<0.001
Pedestrians crossings help walkers feel safe crossing	1.69	1.36	2.03	<0.001
Overhead bridges to cross the road safely	1.76	1.38	2.14	<0.001
Crime safety (Factor 7)				
Neighbourhood streets well lit at night	1 (constrained)			
Walkers and bikers on the streets	0.80	0.61	0.99	<0.001
See and speak to other people when walking	1.11	0.90	1.33	<0.001
High crime rate	−1.39	−1.63	−1.15	<0.001
Crime rate unsafe to go on walks during the day	−1.69	−1.94	−1.45	<0.001
Crime rate unsafe to go on walks during at night	−1.84	−2.11	−1.57	<0.001
Land use diversity (Factor 8)				
Provisions shop	1 (constrained)			
Wet market	1.06	0.83	1.29	<0.001
Supermarket	1.51	1.16	1.87	<0.001
Hardware store	1.95	1.52	2.37	<0.001
Laundry/dry cleaner	0.21	1.52	2.34	<0.001
Clothing	1.89	1.52	2.26	<0.001
Hairdresser	1.53	1.25	1.82	<0.001
Hawker center	1.18	0.94	1.41	<0.001
Coffee shop	1.18	0.96	1.41	<0.001
Fast food restaurant	1.27	0.91	1.64	<0.001
Non-fast food restaurant	1.36	0.98	1.75	<0.001
Bank/Automated Teller Machines (ATM)	1.84	1.49	2.20	<0.001
Post office	1.70	1.31	2.09	<0.001
Library	1.19	0.89	1.50	<0.001
Religious institutions	0.96	0.61	1.30	<0.001
Elementary school	1.66	1.26	2.06	<0.001
Other schools	1.68	1.29	2.07	<0.001
Video store	1.66	1.27	2.04	<0.001
Movie theatre	1.04	0.77	1.31	<0.001
Medicinal shops	1.61	1.31	1.92	<0.001
Clinics/dentals	1.76	1.41	2.11	<0.001
Nearest bus stop	0.76	0.60	0.93	<0.001
Nearest mass rapid transport (MRT) stations	0.85	0.53	1.17	<0.001
Recreational park	1.74	1.33	2.14	<0.001
Community club/residents’ committee	1.88	1.49	2.27	<0.001
Swimming pools	1.79	1.42	2.17	<0.001
Gym or fitness facility	1.48	1.08	1.87	<0.001
Singapore pools	2.14	1.74	2.53	<0.001
Senior activity center/club	1.22	0.90	1.53	<0.001
Book store	1.06	0.75	1.37	<0.001
